# Finding a signal hidden among noise: how can predators overcome camouflage strategies?

**DOI:** 10.1098/rstb.2019.0478

**Published:** 2020-05-18

**Authors:** James A. M. Galloway, Samuel D. Green, Martin Stevens, Laura A. Kelley

**Affiliations:** Centre for Ecology and Conservation, University of Exeter (Penryn Campus), Cornwall TR10 9FE, UK

**Keywords:** camouflage breaking, predator–prey interactions, camouflage, predator vision, cognition

## Abstract

Substantial progress has been made in the past 15 years regarding how prey use a variety of visual camouflage types to exploit both predator visual processing and cognition, including background matching, disruptive coloration, countershading and masquerade. By contrast, much less attention has been paid to how predators might overcome these defences. Such strategies include the evolution of more acute senses, the co-opting of other senses not targeted by camouflage, changes in cognition such as forming search images, and using behaviours that change the relationship between the cryptic individual and the environment or disturb prey and cause movement. Here, we evaluate the methods through which visual camouflage prevents detection and recognition, and discuss if and how predators might evolve, develop or learn counter-adaptations to overcome these.

This article is part of the theme issue ‘Signal detection theory in recognition systems: from evolving models to experimental tests'.

## 1. Introduction

Natural systems are visually noisy environments with each scene comprising a huge variety of colours, textures, shapes and perspectives that must be processed by an observer. Given the vast amount of visual information present in a scene, an observer must attempt to extract ecologically relevant information by reducing the amount of extraneous noise during visual and cognitive processing mechanisms [1]. Animal colour patterns can exploit mechanisms of vision, including pre-existing perceptual biases, to either stand out against the visual background noise (for example to attract a mate) or to blend into it (for example to avoid predation) [2]. Animal coloration has been studied for well over 150 years [3], but advances in experimental methods and new technologies in recent years have more formally applied knowledge of how visual information is processed to reveal new insights into the perception of camouflage from the view of the predator and test how different types of camouflage work [4,5].

Animal camouflage can be thought of as adaptations that act to reduce the likelihood that a target animal is detected and/or recognized by visually hunting predators, through combinations of colour, pattern, morphology and behaviour [4,6]. Note that some camouflage strategies can also work against other modalities (see [7]), but we mostly focus on visual ones here since these have received by far the most attention. A number of distinct strategies that exploit predator vision and cognition in a variety of ways have been identified (table 1; for comprehensive reviews also see [4] and [1]). All strategies exploit some aspect of visual processing and/or cognition and often act to reduce the signal-to-noise ratio (SNR), whereby cues from the camouflaged individual constitute the signal and additional factors that interfere with the true identification of the signal comprise noise. The SNR contrasts the amount of useful information within a visual scene of an observer to that which is either irrelevant or deceptive, and so camouflage can act to reduce the SNR by manipulating the signal, noise or both [8].

**Table 1.** Brief definitions of common camouflage terms discussed in this review (see [4] for more detailed overview).

*Background matching:* general appearance matches the colour, contrast, lightness and pattern of one (specialist) or multiple (generalist) backgrounds.
*Disruptive coloration:* contrasting markings that generate the appearance of false edges within the body surface and/or break up the true body outline to thwart detection or recognition of body shape.
*Countershading:* where coloration of the body surface facing ambient lighting (usually the dorsal surface) is darker than the opposite body surface. This may act to reduce the effect of shadowing from directional lighting (self-shadow concealment) or removing cues of three-dimensional form (obliterative shading).
*Transparency:* body tissues rendered colourless owing to lack of pigment expression, preventing detection. This may involve the entire body volume (common in many pelagic invertebrates) or discrete body panels (as displayed in Lepidoptera spp).
*Masquerade:* hindering recognition after detection by resembling an uninteresting or inedible object from within the environment, such as a stick or leaf.
*Distractive markings:* colour patches or patterns that draw the attention of observer away from cues such as body outline that would facilitate object detection.

Since predator vision and cognition impose selection on the evolution of camouflage [9], the importance of incorporating an understanding of predator perception into the quantification and analysis of camouflage has been widely recognized for many years [10]. If the defensive coloration of prey has been under selection to exploit weaknesses within predator cognitive and visual processes, then it is likely that selection has also acted on predator perception to increase the camouflage-breaking capacity of the cognitive and visual processes themselves. While we have made significant advances in understanding how camouflage acts to exploit predator cognition and visual processing [2,8,9,11,12], there has been comparably less recent empirical investigation of the predators themselves and what camouflage-breaking counter-adaptations may have arisen owing to the selective pressures imposed on the predator by the need to find camouflaged prey. Several decades ago, work focused predominantly on predator behaviours such as search rates and aspects of attention in finding hidden prey (see [13,14] for discussion) but neglected how camouflage itself works and the types of camouflage strategy that exist (i.e. prey defences). Here, we bring these various avenues of research together. We briefly review recent advances in how camouflage prevents detection and recognition, discuss if and how predators might overcome these strategies and conclude by identifying several areas for future research.

## 2. Camouflage strategies

In recent years, there have been several thorough reviews synthesizing the field of animal camouflage and underlying mechanisms (for example: [1,4,8]). Here, we introduce and discuss how certain camouflage strategies act against predator vision and cognition with reference to recent additions to the literature to minimize cross-over with these earlier works. In doing so, we set the scene to discuss potential predator counter-adaptations.

### (a) The signal

Background matching (table 1) acts to reduce the SNR by concealing the body's appearance within the animal's background. Perhaps the most quintessential camouflage strategy, it is found across a variety of taxa, including the classically studied peppered moth (*Biston betularia*) [15,16], as well as multiple other species in terrestrial [17] and aquatic environments [18–20]. This strategy can be limiting as backgrounds in nature are rarely uniform, and the camouflaged individual loses the selective benefits of coloration if it leaves its ‘matched patch' [21,22]. There are multiple solutions that may overcome this, including optimizing camouflage so that it works effectively across multiple visual scenes (see review by [23]), and using behaviour to choose appropriate substrates [6,24], such that background matching can still be an effective camouflage strategy [16,25]. However, regardless of how perfect background matching may be in terms of disguising the body surface, the outline of the body (i.e. the edges) can provide predators with a cue to home in on [26,27].

The edges of objects have a crucial role in visual processing, aiding object detection and recognition by revealing the boundaries between objects and between objects and their backgrounds [8,28,29]. Disruptive coloration (table 1) manipulates the SNR and can exploit different mechanisms of visual processing. It can act to minimize the signal by combining camouflage-matched colours (differential blending) with markings breaking up the true body edges, while also creating noise through contrasting colour patches that create the appearance of false edges over an animal's body surface that are more salient than true edges (maximum disrupted contrast) [28–30]. In doing so, it can exploit the way that visual systems encode edges and boundaries [31,32]. While it can hinder detection by naïve observers, disruptive coloration has also been shown to hinder camouflage-breaking capabilities of predators that have some degree of familiarity with prey. This includes by impeding search image formation (a temporary increase in attention of predators towards specific prey phenotypes) and switching to new camouflage types, even with increasing experience [30] (discussed in §3c). Disruptive patterning is also commonly associated with edge enhancement, where dark markings have darker edges and light markings have lighter edges [28]. Edge-enhanced markings may benefit a camouflaged target by masking salient features, but they may also create false depth cues that impair shape discrimination and recognition [28,29,33].

Another difficulty that must be overcome to improve camouflage is to counteract shadowing from directional lighting. Countershading (table 1) acts to reduce the SNR by obliterating cues to three-dimensional shape via self-shadow concealment and/or by enhancing the level of background matching [34,35]. The effectiveness of countershading is highly dependent on the orientation of the animal and the surrounding lighting conditions [36–38]. This ‘illumination-dependence' of countershading has been further tested with humans in simulated three-dimensional environments, where visual search was slower and less accurate when the target's countershading was optimal for the lighting conditions of the scene [34]. Animals from aquatic environments often display two-tone coloration analogous to that observed in countershading terrestrial species. In these conditions, countershading may reduce the SNR from multiple angles providing directional background matching in aquatic environments with the lighter ventral surface being viewed against downwelling irradiance and the darker ventral surface viewed against deeper water [39,40].

Transparency is conceptually perhaps the ideal form of camouflage since an animal's body would provide a window through to its background, providing mobile and accurate camouflage. However, while the absence of pigment minimizes surface radiance, the tissues themselves must also minimize the scattering of light and inevitably some organs will persist that could attract predator attention [41–43]. Transparency acts to minimize the SNR by reducing the body silhouette while simultaneously potentially allowing noise from the environment to transverse the body, allowing for camouflage in open environments where there are no surfaces to match. Therefore, it is unsurprising that transparency is most strongly represented in pelagic communities, facilitated by the fact that water is closer in refractive index to many biological constituents compared to air, reducing scattering [41]. However, there are terrestrial exceptions: for example, many Ithomiine butterflies possess transparent or partially transparent wings [44]. Transparency enhances crypsis to both avian and human predators by reducing the chance of detection compared to more opaque species [44]. Similarly, in a recent study, the survival of cryptic moths with wings that include transparent windows was comparable to wingless bodies but significantly higher than opaque cryptic forms, suggesting that transparency is also a suitable antipredator strategy for terrestrial prey [45].

The camouflage strategies discussed so far act to reduce the likelihood of detection by predator vision, but predator cognition is also intrinsically linked to the evolution of camouflage [9]. Masquerade (table 1) exploits predator cognition, promoting misclassification by increasing noise via a false signal [8]. Conceptually, the key difference between crypsis and masquerade is that the former is dependent on avoiding detection, whereas the latter involves being detected but then misidentified as an inedible or unimportant feature of the environment. Masquerade has been predominantly studied in caterpillars that masquerade as twigs [46–48], but has recently been demonstrated in animals that can dynamically change their appearance. The European cuttlefish *Sepia officinalis* appears to be able to modify skin colour and texture to resemble uniformly coloured smooth rocks or mottled irregularly shaped rocks [49]. However, demonstrating that the intended receiver perceives masquerading cuttlefish as inanimate rocks, as opposed to blending into a rock background, is challenging.

### (b) The environment

A core prediction for the success of most types of camouflage is that there will be close associations between an animal's phenotype and elements of its environment. This may also influence the type of camouflage that predominates. For example, juvenile shore crabs *Carcinus maenas* live in a range of intertidal habitats and exhibit facultative habitat-specific and ontogenetic camouflage strategies. Crabs in visually complex rock pool habitats display high variation among individuals and increased levels of disruptive coloration, whereas crabs in more visually uniform mudflat habitats show less variation and rely on matching the colour, pattern and brightness of their backgrounds [22,50]. Furthermore, crabs also change their own appearance with age, likely linked to changes in habitat use and camouflage strategy [51].

Cryptic species face a significant constraint in that generally motion breaks camouflage [1]. However, the complexity of a visual environment can be temporal as well as spatial, and motion such as wind-blown foliage and photic changes in illumination is ubiquitous in natural environments [52–54]. Species may be able to capitalize on large amounts of dynamic noise within the visual environment to mask movement. Behaviour can facilitate camouflage of moving objects. For example, Macleay's spectre stick insects *Extatosoma tiaratum* use environmental cues to adjust their swaying behaviour in response to wind, resulting in motion that is quantitatively similar to wind-blown vegetation [55]. Environments containing dynamic illumination such as dappled light and water caustics can also act as potential movement refuges owing to the large amount of background noise [52,53]. Dappled lighting appears to disrupt motion detection capabilities, as search efficiency is worse in simulated dappled lighting and water caustics compared to static scenes [53] and it increases latency to identify and target prey [56]. Overall, these studies demonstrate that dynamic noise can mask movement and that animals may be able to decrease their SNR through temporal background matching via movement or seeking out environments with more dynamic noise.

## 3. Predator strategies

**Figure 1.**
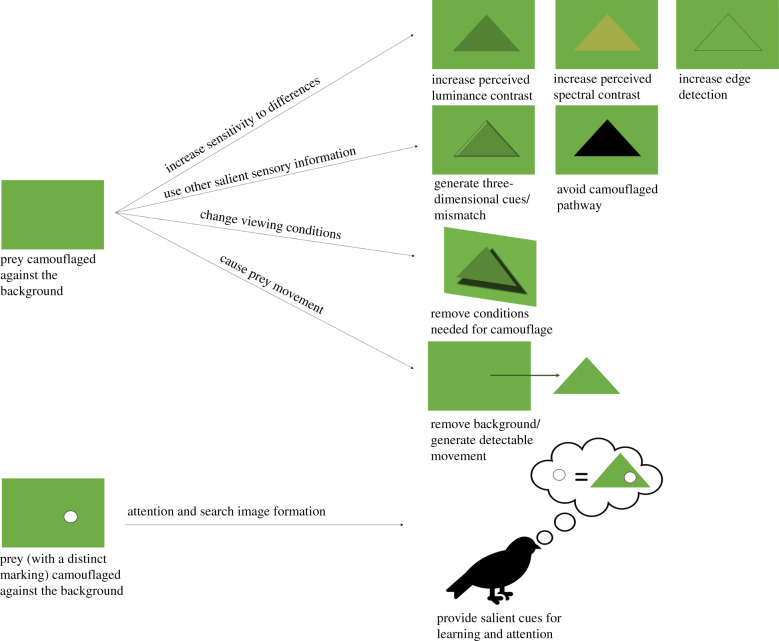
A graphical representation of the potential effects of predator strategies for overcoming camouflage on prey crypsis. Hypothetical backgrounds represented as green rectangles, and prey as green triangles.

As previously discussed, camouflage can be described as reducing the cues from a given animal (signal) to better blend in with visual information from the environment (noise), or those that create false signals. However, how predators overcome camouflage strategies has received far less study. Logically, predators must directly oppose the reduction in SNR in order to successfully locate and/or identify prey, with one way to do so being to widen the difference between the signal of the prey species and the environmental noise via selection for greater sensitivity in relevant senses. Alternatives also include using other sensory modalities, either in concert with, or instead of the originally targeted senses. Finally, predators could adjust behaviours to improve searching methods, improve perceptual filtering methods and alter attention by changing the environmental context that camouflage depends on (such as viewing angle or lighting condition, figure 1), or flush prey to cause movement and increase conspicuousness. Here, we describe some of what is known or can be inferred about predator strategies for overcoming camouflage.

### (a) Improvements in the sensory pathways involved in prey detection

By increasing sensitivity or selecting for characteristics of vision that are better suited for the task, predators could widen the SNR to increase the likelihood of detection, recognition and subsequent capture. Early discussions and work proposed that dichromatic animals may be better able to break colour camouflage since they could more readily detect luminance and contrast differences and be less affected by colour noise. Initial evidence supported this; reduced colour sensitivity aiding camouflage discrimination was suggested in humans where, for example, the lack of colour information may aid object recognition where colour patches would otherwise obscure an object's outline [57]. Further research supported this idea, showing how humans with colour vision deficiency outperform trichromatic individuals in searching for camouflaged targets where colour is irrelevant to the task (i.e. colour adds noise when differentiating textures) [58,59]. However, in these studies, the stimuli were highly artificial with little analogy to natural cryptic systems. Beyond humans, there is evidence in vision-polymorphic primates that while trichromats are ultimately better at finding prey, dichromatic individuals found camouflaged prey more often, especially in situations where colour is less important or light levels are low [60,61]. However, these and other studies tend to be indirect/correlative and do not experimentally test for differences in the detection of camouflaged targets with different visual systems. In more recent computer experiments, however, trichromatic humans searching for camouflaged birds showed an overall advantage in finding cryptically coloured prey over simulated dichromatic conditions, although dichromats were less affected by luminance conditions [62]. Trichromats may be better able to discriminate between the colours of the prey and substrate at any given time. By contrast, dichromats may be less affected by chromatic noise, especially at low light levels, that otherwise may obscure textural or achromatic differences between prey and substrate. Overall, we can assume prey camouflage could play a role in the selection for predator colour vision.

Spatial/contrast acuity also varies between species, and greater acuity may help in breaking camouflage. A significant part of object recognition is the identification of shapes and outlines [8], and predators with better acuity should be better able to perceive these cues, as well as differences between masqueraders and the objects they match. They also should be better able to overcome the effects of camouflage types like background matching and disruptive camouflage [63], as the increased sensitivity to spatial differences should compensate for the reduction in the clarity of edge cue. Another factor to consider is distance-dependent camouflage, which relies on the limitations of predator acuity to obscure patterns, allowing prey to camouflage when viewed from a distance [2,64]. Predators with high spatial acuity may be able to resolve these patterns (and any other salient features) and locate prey. However, the role of visual acuity in breaking camouflage requires further exploration.

Predators could also evolve systems that obtain more complex information that may make it harder for prey to maintain camouflage. Stereopsis has been demonstrated in a variety of taxa (reviewed in [65], see also [66,67]). Using the differences in information from each eye, three-dimensional structures can be resolved, providing an additional cue in prey detection and recognition. This means that prey have an additional characteristic they must hide in order to prevent detection. Binocular/static disparity has been shown to reduce the effectiveness of disruptive markings with edge enhancement, by clarifying the true three-dimensional structure of the camouflaged object [68], although it is less effective when faced with moving objects [69]. This is likely owing to edge enhancement causing artificial depth, which is revealed when binocular disparity identifies the true three-dimensional shape of the object. Motion parallax—the differential shift in the relative positions of prey and substrates during predator movement—may also provide these cues but this does require predator movement to generate the shift [68]. Other higher level cognitive processes may also play a role in perceiving prey movement when camouflaged. Praying mantises (*Sphodromantis lineola*) use second-order cues to detect the movement of prey that were otherwise matching the average luminance of the background, potentially overcoming the camouflage of first-order motion cues, such as changes in luminance over time [70].

### (b) Using additional sensory pathways to the primary modality underlying camouflage

Predators may use a specific region within a sensory modality in which animals are not camouflaged. For example, a variety of species exhibit spectral sensitivity that extends into the ultraviolet (UV) region of the light spectrum, something invisible to humans [71]. There are examples of animals that are cryptic in the visible spectrum but are non-matching in the UV region [72,73], and UV-sensitive predators could have an advantage in detecting them [74]. Alternatively, predators can use other physical properties of light; for example, cuttlefish are sensitive to polarized light, which may break the silvering/countershading camouflage of fish, as individuals preferentially attack fish that reflected linearly polarized light [75]. While the effectiveness of polarization vision at longer ranges is limited [76], at closer range, it could aid in defeating the luminance and chromatic camouflage of countershading/silvering [77], and also potentially of transparency.

Less well studied are examples where entirely separate modalities help in breaking camouflage. Given human reliance on vision, the concept of camouflage has mostly been focused on visual crypsis and less is known generally about other signalling modalities as a result (but see [7]). Chemical cues can attract predators to prey location [78], and multiple species use auditory cues in prey location and capture [79], but there is little empirical testing of their effect on predation of camouflaged prey. Many elasmobranch species are capable of using weak electrochemical cues to locate prey [80] that are often cryptic, such as flatfish [81]. While it is evident that electroreception aids in overcoming prey camouflage, it is unclear if it evolved in response to prey crypsis. The complexity lies in identifying whether these sensory systems have been co-opted from another primary purpose in response to camouflaged prey or have evolved as the primary sensory system for predation.

### (c) Changes in cognitive processes and behaviour

Search images, where predators use previous experience of prey to improve search efficiency by focusing attention on salient features, have been a concept in behavioural ecology and psychology for more than half a century [82]. Predators can improve their ability to find prey by focusing on specific features, specializing in detecting and recognizing them, even if the prey are camouflaged, especially if these features are common in the prey population. In a situation with multiple cryptic species or morphs within a species, predators show an improved ability to locate and capture the more common prey, based on the increased experience in locating and handling those individuals [83]. When prey phenotypes could evolve in response to predation, they showed increased phenotypic variation compared to control non-evolved prey, suggesting selection for disruption of the predator search image. While this is one example in systems relevant to camouflage, multiple other studies provide evidence of search image formation [84,85]. As discussed above, camouflage strategies such as disruptive patterns can interfere with search image formation [30], whereas conspicuous ‘distractive' markings thought to draw attention away from the prey outline reduced prey survival [86,87], perhaps by providing distinctive markings used in search image formation. Predators that can learn to recognize key features of camouflage may be better able to locate them in the future, increasing the likelihood of prey detection because of the camouflage meant to hide them. Learning has also been shown to be effective in overcoming masquerade, based on the size disparity between mimics and models [47].

As part of searching behaviour, the viewing conditions of the predator may also have an impact on camouflage efficacy. Prey orientation relative to resting substrate has a significant effect on the effectiveness of various camouflage strategies [6,24,37]. Taking countershading as an example, there is experimental evidence that the effectiveness of countershading is dependent on the illumination conditions of prey [34]. It relies on the strength of countershading being appropriate to the strength and directionality of illumination. Predators could search at specific times of days or under weather conditions that reduce the effectiveness of countershading and crypsis in general. Orientation relative to observers is also important, with deviations from an optimum viewing angle by as little as 15° resulting in increased detection speed and accuracy [88]. As predators move through the environment while searching for prey, the orientation of prey relative to both the predator and any illumination is unlikely to be consistently optimal, which could increase the likelihood of successful detection, recognition and capture. Types of predator that are particularly mobile in three dimensions, such as birds, could move up or down in the habitat to search for prey from below, side on and above, such that certain viewpoints may reveal prey better. In the aquatic environment, predators can also move freely in the water, circling around possible prey locations to detect targets [89]. While countershading may improve background matching and remove shape from shading cues, in many terrestrial animals, the animal itself will still cast a shadow on the ground that can be used as a cue by predators. Prey orientation, strength and direction of illumination and predator location will all affect the presence and strength of these cues, but the role of ground shadows in breaking camouflage has received little attention. Habitat selection could also play a role in defeating camouflage, with predators potentially searching in more uniform backgrounds to improve target detection. Background complexity is known to facilitate camouflage [90,91] and so predators should benefit from focusing their efforts on patches of lower complexity.

Finally, perhaps the simplest way to overcome camouflage is to bypass it entirely. Motion has been shown to break, or at least significantly limit the efficacy of camouflage [8,92,93]. If predators manage to disturb prey sufficiently, then they can move them off matching substrates and widen the SNR and improve detection. Painted redstarts (*Myioborus pictus*), for example, perform a series of stereotypical behaviours involving body pivoting coupled with wing and tail spreading, and high contrast colours. These elicit a flush response from prey that can disturb cryptic species from matching resting substrates, increasing detection and capture [94,95]. Although prey species vary in their propensity to flush depending on their defensive strategies, with cryptic prey less likely to flush (81%) compared to structural (95%) or aposematic (90%) defended species, the overall high response rate suggests this is an effective way to overcome camouflage [96].

## 4. Future directions

### (a) Identifying other forms of camouflage-breaking adaptations

We have proposed only a handful of potential anti-camouflage strategies, and there are undoubtedly other strategies that the sensory and cognitive systems of predators have evolved to overcome camouflage. How does the tuning of sensory systems affect detection success? Animals have demonstrated a variety of ways of focusing aspects of senses to specific tasks: do these adaptations aid in the improvement of prey detection, and did they evolve in response to camouflage strategies, or were they merely co-opted into that task? These and other adaptations need to be investigated for their potential effect on camouflage.

### (b) Breaking camouflage in other sensory modalities

Camouflage is generally considered in terms of visual concealment, but animals also mask other sensory cues that this review has not discussed. There are increasing examples of animals camouflaging themselves via other sensory modalities (reviewed in [7]); for example, moths use auditory camouflage to avoid detection by bats [97]. In response, some bats appear to use textural cues from acoustic differences between moths and substrates to overcome this camouflage and locate prey [79]. However, our understanding of camouflage in non-visual modalities is generally lacking and focus on camouflage and anti-camouflage strategies in other sensory modalities is needed. The same approach taken in modern studies of camouflage should be applied, where differences between environmental noise and prey signal are quantified relative to predator senses, allowing for the formation of measures of detectability.

### (c) Evolutionary history of predators and sensory systems

As discussed above, it is likely that predators will not rely on a single sense when detecting prey. How multiple senses interact, whether they are integrated or hierarchical, how they are differentially selected for and how important they are, are all questions that must be answered in order to fully understand how predators overcome camouflage. Empirical tests that model predator effectiveness with different combinations of senses available to them could start identifying each sense's priority based on how its removal affects prey capture success. Understanding the evolutionary history of predator senses and prey camouflage strategies could also give an insight into the influence they have on each other and how new strategies might arise. If there are links between the use of specific senses to overcome specific camouflage strategies, we would expect to see these associations reflected in the evolutionary history of the species. As prey generally evolve defences that are effective against a wide range of predators they face, we might predict that predator strategies to overcome these defences are also generalist. It is also worth considering the asymmetrical selective pressures facing predators and prey. The life-dinner hypothesis [98] indicates that there should be a greater pressure on camouflage to defeat predator detection, given the higher costs of it failing: death of the prey versus a missed meal for the predator. An additional related factor is that many predators will not solely depend on one prey species and may simply switch to targeting other non-camouflaged prey when their success is low. This will further influence the degree of selection on coevolution between prey defences and predator counter-adaptations. Targeting specific predator–prey dyads that have co-evolved would allow us to identify how prey evolve new strategies to avoid predators and how predators respond.

### (d) Empirical testing of different camouflage strategies and predator strategies to overcome them

Much of what we have proposed about the potential mechanisms predators use to break camouflage (figure 1) are at best based on inferences from research on what conditions best favour camouflage. What is needed moving forward is to continue directly testing the effectiveness of anti-camouflage strategies, either with real predators or with human participants and modified stimuli to mimic realistic predator vision. These methods can be used to directly test variation in prey capture success in real/artificial predators with, for example, manipulated acuity. Are predators with poor visual acuity able to overcome texture/pattern-based markings similar to colour in dichromat versus trichromat? Or do camouflage strategies shift with predator acuity, for example, with high acuity selecting for disruptive markings, and low acuity selecting for background matching?

### (e) Further investigation of the role of cognition in breaking camouflage

Evolutionary change in visual systems may take significantly more time than the development of cognitive flexibility or changes in attention-based behaviours. Consequently, there may be a greater diversity and abundance of cognitive adaptations to break camouflage, especially as these are likely to be much more flexible in the short term. While predator cognition in relation to breaking camouflage is still somewhat limited to the examples discussed in this review, more work has been done on how Batesian mimics (where an undefended prey resembles a defended prey) exploit the cognitive processes of their predators to avoid predation [99,100]. As with masquerade, quantifying the sensory and cognitive processes involved in misclassification is more straightforward because we can identify what perfect resemblance (i.e. the model) looks like, and manipulate various aspects of prey appearance and predator experience to quantify when misclassification breaks down. Although a similar approach is more challenging for cryptic prey, we can now more accurately quantify and account for predator perception, which will allow us to begin to identify when cognitive processes come into play.

Senses perform a variety of functions, and the cost of focusing on one task over another could be the reduction in efficacy of the latter. In a hypothetical trade-off, changing features of vision to focus more on camouflage than other tasks could limit other visually guided behaviours such as navigation. Cognitive and behavioural adaptations may allow predators to break camouflage without compromising other sensory functions, and their relative benefits compared to physical adaptations should be investigated further.

## 5. Concluding remarks

There is a significant and ever-growing body of literature surrounding the diversity, mechanisms, functions and evolution of camouflage. Increasingly its success is quantified based on the sensory systems of relevant predators targeted by camouflage. However, there is a lack of empirical testing of how predators overcome camouflage. This is likely owing to the relative ease of identifying the impacts of selection imposed by predation, where changes in morphological characteristics and phenotypes can be quantified. It is more complex, but still necessary, to identify the changes in the visual systems, cognition and behaviour of predators. Just as any one species often uses multiple forms of crypsis, it is likely that their predators have multiple means of overcoming them, in response to multiple prey types. The next steps should be to directly investigate these camouflage-breaking adaptations, both to identify them and characterize their relationship to, and effectiveness against camouflage strategies. If possible, phylogenetic history could be investigated to identify the selective pressures acting on camouflage and its opponents, and whether one imposes a greater selective pressure upon the other.

## Data accessibility

This article has no additional data.

## References

[1] Cuthill IC 2019 Camouflage. J. Zool. 308, 75–92. (10.1111/jzo.12682)

[2] Stevens M 2007 Predator perception and the interrelation between different forms of protective coloration. Proc. R. Soc. B 274, 1457–1464. (10.1098/rspb.2007.0220) PMC1950298 17426012

[3] Wallace AR 1889 Darwinism: An exposition of the theory of natural selection, with some of its applications. Cambridge, UK: Cambridge University Press.

[4] Stevens M, Merilaita S 2009 Animal camouflage: current issues and new perspectives. Phil. Trans. R. Soc. B 364, 423–427. (10.1098/rstb.2008.0217) 18990674 PMC2674078

[5] Cuthill IC et al. 2017 The biology of color. Science 357, eaan0221 (10.1126/science.aan0221) 28774901

[6] Stevens M, Ruxton GD 2018 The key role of behaviour in animal camouflage. Biol. Rev. 94, 116–134. (10.1111/brv.12438) PMC6378595 29927061

[7] Ruxton GD 2009 Non-visual crypsis: a review of the empirical evidence for camouflage to senses other than vision. Phil. Trans. R. Soc. B 364, 549–557. (10.1098/rstb.2008.0228) 19000976 PMC2674081

[8] Merilaita S, Scott-Samuel NE, Cuthill IC 2017 How camouflage works. Phil. Trans. R. Soc. B 372, 20160341 (10.1098/rstb.2016.0341) 28533458 PMC5444062

[9] Skelhorn J, Rowe C 2016 Cognition and the evolution of camouflage. Proc. R. Soc. B 283, 20152890 (10.1098/rspb.2015.2890) PMC4810834 26911959

[10] Endler JA 1978 A predator's view of animal color patterns. In Evolutionary biology (eds Hecht MK, Steere WC, Wallace B), pp. 319–364. Boston, MA: Springer US.

[11] Endler JA, Mappes J 2017 The current and future state of animal coloration research. Phil. Trans. R. Soc. B 372, 20160352 (10.1098/rstb.2016.0352) 28533467 PMC5444071

[12] Caves EM, Johnsen S 2018 AcuityView: an r package for portraying the effects of visual acuity on scenes observed by an animal. Methods Ecol. Evol. 9, 793–797. (10.1111/2041-210X.12911)

[13] Bond AB 2007 The evolution of color polymorphism: crypticity, searching images, and apostatic selection. Annu. Rev. Ecol. Evol. Syst. 38, 489–514. (10.1146/annurev.ecolsys.38.091206.095728)

[14] Ruxton GD, Allen WL, Sherratt TN, Speed MP 2018 Avoiding attack: the evolutionary ecology of crypsis, aposematism, and mimicry, 2nd edn Oxford, UK: Oxford University Press.

[15] Kettlewell HBD 1955 Selection experiments on industrial melanism in the Lepidoptera. Heredity 9, 323–342. (10.1038/hdy.1955.36)

[16] Walton OC, Stevens M 2018 Avian vision models and field experiments determine the survival value of peppered moth camouflage. Commun. Biol. 1, 1–7. (10.1038/s42003-018-0126-3) 30271998 PMC6123793

[17] Stevens M, Troscianko J, Wilson-Aggarwal JK, Spottiswoode CN 2017 Improvement of individual camouflage through background choice in ground-nesting birds. Nat. Ecol. Evol. 1, 1325–1333. (10.1038/s41559-017-0256-x) 28890937 PMC5584661

[18] Buresch KC, Maethger LM, Allen JJ, Bennice C, Smith N, Schram J, Chiao C-C, Chubb C, Hanlon RT 2011 The use of background matching vs. masquerade for camouflage in cuttlefish *Sepia officinalis*. Vision Res. 51, 2362–2368. (10.1016/j.visres.2011.09.009) 21964504

[19] Clarke JM, Schulter D 2011 Colour plasticity and background matching in a threespine stickleback species pair. Biol. J. Linn. Soc. 102, 902–914. (10.1111/j.1095-8312.2011.01623.x)

[20] Siegenthaler A, Mastin A, Dufaut C, Mondal D, Benvenuto C 2018 Background matching in the brown shrimp *Crangon crangon*: adaptive camouflage and behavioural-plasticity. Sci. Rep. 8, 3292 (10.1038/s41598-018-21412-y) 29459624 PMC5818513

[21] Green SD, Duarte RC, Kellett E, Alagaratnam N, Stevens M 2019 Colour change and behavioural choice facilitate chameleon prawn camouflage against different seaweed backgrounds. Commun. Biol. 2, 1–10. (10.1038/s42003-019-0465-8) 31263774 PMC6588621

[22] Price N, Green S, Troscianko J, Tregenza T, Stevens M 2019 Background matching and disruptive coloration as habitat-specific strategies for camouflage. Sci. Rep. 9, 1–10. (10.1038/s41598-019-44349-2) 31127182 PMC6534618

[23] Hughes A, Liggins E, Stevens M 2019 Imperfect camouflage: how to hide in a variable world? Proc. R. Soc. B 286, 20190646 (10.1098/rspb.2019.0646) PMC6532520 31088268

[24] Kang C-K, Moon J-Y, Lee S-I, Jablonski PG 2012 Camouflage through an active choice of a resting spot and body orientation in moths. J. Evol. Biol. 25, 1695–1702. (10.1111/j.1420-9101.2012.02557.x) 22775528

[25] Michalis C, Scott-Samuel NE, Gibson DP, Cuthill IC 2017 Optimal background matching camouflage. Proc. R. Soc. B 284, 20170709 (10.1098/rspb.2017.0709) PMC5524497 28701559

[26] Cott HB 1940 Adaptive coloration in animals. London, UK: Methuen.

[27] Thayer GH, Thayer AH 1909 Concealing-coloration in the animal kingdom; an exposition of the laws of disguise through color and pattern: being a summary of Abbott H. Thayer's discoveries. New York, NY: The Macmillan Co See https://www.biodiversitylibrary.org/item/118302.

[28] Sharman RJ, Lovell PG 2019 Edge-enhanced disruptive camouflage impairs shape discrimination. Percept. 10, 1–9. (10.1177/2041669519877435) PMC6749785 31555431

[29] Sharman RJ, Moncrieff SJ, Lovell PG 2018 Dissociating the effect of disruptive colouration on localisation and identification of camouflaged targets. Sci. Rep. 8, 1–6. (10.1038/s41598-018-25014-6) 29700366 PMC5920097

[30] Troscianko J, Skelhorn J, Stevens M 2018 Camouflage strategies interfere differently with observer search images. Proc. R. Soc. B 285, 20181386 (10.1098/rspb.2018.1386) PMC6158535 30185636

[31] Osorio D, Srinivasan MV, Pettigrew JD 1991 Camouflage by edge enhancement in animal coloration patterns and its implications for visual mechanisms. Proc. R. Soc. B 244, 81–85. (10.1098/rspb.1991.0054) 1679552

[32] Stevens M, Cuthill IC 2006 Disruptive coloration, crypsis and edge detection in early visual processing. Proc. R. Soc. B 273, 2141–2147. (10.1098/rspb.2006.3556) PMC1635512 16901833

[33] Egan J, Sharman RJ, Scott-Brown KC, Lovell PG 2016 Edge enhancement improves disruptive camouflage by emphasising false edges and creating pictorial relief. Sci. Rep. 6, 1–9. (10.1038/srep38274) 27922058 PMC5138594

[34] Penacchio O, Lovell PG, Harris JM 2018 Is countershading camouflage robust to lighting change due to weather? R. Soc. Open Sci. 5, 170801 (10.1098/rsos.170801) 29515822 PMC5830711

[35] Rowland HM, Cuthill IC, Harvey IF, Speed MP, Ruxton GD 2008 Can't tell the caterpillars from the trees: countershading enhances survival in a woodland. Proc. R. Soc. B 275, 2539–2545. (10.1098/rspb.2008.0812) PMC2605806 18700207

[36] Cuthill IC, Sanghera NS, Penacchio O, Lovell PG, Ruxton GD, Harris JM 2016 Optimizing countershading camouflage. Proc. Natl Acad. Sci. USA 113, 13 093–13 097. (10.1073/pnas.1611589113) PMC5135326 27807134

[37] Penacchio O, Cuthill IC, Lovell PG, Ruxton GD, Harris JM 2015 Orientation to the sun by animals and its interaction with crypsis. Funct. Ecol. 29, 1165–1177. (10.1111/1365-2435.12481) 26937063 PMC4758631

[38] Penacchio O, Lovell PG, Cuthill IC, Ruxton GD, Harris JM 2015 Three-dimensional camouflage: exploiting photons to conceal form. Am. Nat. 186, 553–563. (10.1086/682570) 26655578

[39] Kelley JL, Merilaita S 2015 Testing the role of background matching and self-shadow concealment in explaining countershading coloration in wild-caught rainbowfish. Biol. J. Linn. Soc. 114, 915–928. (10.1111/bij.12451)

[40] Kelley JL, Taylor I, Hart NS, Partridge JC 2017 Aquatic prey use countershading camouflage to match the visual background. Behav. Ecol. 28, 1314–1322. (10.1093/beheco/arx093)

[41] Bagge LE 2019 Not as clear as it may appear: challenges associated with transparent camouflage in the ocean. Integr. Comp. Biol. 59, 1653–1663. (10.1093/icb/icz066) 31141119

[42] Cronin TW 2016 Camouflage: being invisible in the open ocean. Curr. Biol. 26, R1179–R1181. (10.1016/j.cub.2016.09.056) 27875694

[43] Johnsen S 2001 Hidden in plain sight: the ecology and physiology of organismal transparency. Biol. Bull. 201, 301–318. (10.2307/1543609) 11751243

[44] Arias M, Mappes J, Desbois C, Gordon S, McClure M, Elias M, Nokelainen O, Gomez D 2019 Transparency reduces predator detection in mimetic clearwing butterflies. Funct. Ecol. 33, 1110–1119. (10.1111/1365-2435.13315)

[45] Arias M, Elias M, Andraud C, Berthier S, Gomez D 2019 Transparency improves concealment in cryptically coloured moths. J. Evol. Biol. 33, 247–252. (10.1111/jeb.13560) 31643116

[46] Skelhorn J 2015 Masquerade. Curr. Biol. 25, R643–R644. (10.1016/j.cub.2015.02.069) 26241133

[47] Skelhorn J, Ruxton GD 2013 Size-dependent microhabitat selection by masquerading prey. Behav. Ecol. 24, 89–97. (10.1093/beheco/ars139)

[48] Skelhorn J, Rowland HM, Speed MP, Ruxton GD 2010 Masquerade: camouflage without crypsis. Science 327, 51 (10.1126/science.1181931) 20044568

[49] Panetta D, Buresch K, Hanlon RT 2017 Dynamic masquerade with morphing three-dimensional skin in cuttlefish. Biol. Lett. 13, 20170070 (10.1098/rsbl.2017.0070) 28356412 PMC5377043

[50] Stevens M, Lown AE, Wood LE 2014 Camouflage and individual variation in shore crabs (*Carcinus maenas*) from different habitats. PLoS ONE 9, e115586 (10.1371/journal.pone.0115586) 25551233 PMC4281232

[51] Nokelainen O, Maynes R, Mynott S, Price N, Stevens M 2019 Improved camouflage through ontogenetic colour change confers reduced detection risk in shore crabs. Funct. Ecol. 33, 654–669. (10.1111/1365-2435.13280) 31217655 PMC6559319

[52] Cuthill IC, Matchette SR, Scott-Samuel NE 2019 Camouflage in a dynamic world. Curr. Opin. Behav. Sci. 30, 109–115. (10.1016/j.cobeha.2019.07.007)

[53] Matchette SR, Cuthill IC, Scott-Samuel NE 2018 Concealment in a dynamic world: dappled light and caustics mask movement. Anim. Behav. 143, 51–57. (10.1016/j.anbehav.2018.07.003)

[54] Peters RA, Hemmi JM, Zeil J 2007 Signaling against the wind: modifying motion-signal structure in response to increased noise. Curr. Biol. 17, 1231–1234. (10.1016/j.cub.2007.06.035) 17614279

[55] Bian X, Elgar MA, Peters RA 2016 The swaying behavior of *Extatosoma tiaratum*: motion camouflage in a stick insect? Behav. Ecol. 27, 83–92. (10.1093/beheco/arv125)

[56] Matchette SR, Cuthill IC, Scott-Samuel NE 2019 Dappled light disrupts prey detection by masking movement. Anim. Behav. 155, 89–95. (10.1016/j.anbehav.2019.07.006)

[57] Anon 1940 Colour-blindness and camouflage. Nature 146, 226 (10.1038/146226a0)

[58] Morgan MJ, Adam A, Mollon JD 1992 Dichromats detect colour-camouflaged objects that are not detected by trichromats. Proc. R. Soc. B 248, 291–295. (10.1098/rspb.1992.0074) 1354367

[59] Saito A, Mikami A, Hosokawa T, Hasegawa T 2006 Advantage of dichromats over trichromats in discrimination of color-camouflaged stimuli in humans. Percept. Mot. Skills 102, 3–12. (10.2466/pms.102.1.3-12) 16671590

[60] Melin AD, Fedigan LM, Hiramatsu C, Sendall CL, Kawamura S 2007 Effects of colour vision phenotype on insect capture by a free-ranging population of white-faced capuchins, *Cebus capucinus*. Anim. Behav. 73, 205–214. (10.1016/J.ANBEHAV.2006.07.003)

[61] Smith AC, Surridge AK, Prescott MJ, Osorio D, Mundy NI, Buchanan-Smith HM 2012 Effect of colour vision status on insect prey capture efficiency of captive and wild tamarins (*Saguinus* spp.) Anim. Behav. 83, 479–486. (10.1016/j.anbehav.2011.11.023)

[62] Troscianko J, Wilson-Aggarwal J, Griffiths D, Spottiswoode CN, Stevens M 2017 Relative advantages of dichromatic and trichromatic color vision in camouflage breaking. Behav. Ecol. 28, 556–564. (10.1093/beheco/arw185) 29622920 PMC5873837

[63] Barnett JB, Cuthill IC, Scott-Samuel NE 2018 Distance-dependent aposematism and camouflage in the cinnabar moth caterpillar (*Tyria jacobaeae*, Erebidae). R. Soc. Open Sci. 5, 171396 (10.1098/rsos.171396) 29515858 PMC5830747

[64] Marshall NJ 2000 Communication and camouflage with the same ‘bright’ colours in reef fishes. Phil. Trans. R. Soc. B 355, 1243–1248. (10.1098/rstb.2000.0676) 11079407 PMC1692842

[65] Nityananda V, Read JCA 2017 Stereopsis in animals: evolution, function and mechanisms. J. Exp. Biol. 220, 2502–2512. (10.1242/jeb.143883) 28724702 PMC5536890

[66] Nityananda V, Tarawneh G, Henriksen S, Umeton D, Simmons A, Read JCA 2018 A novel form of stereo vision in the praying mantis. Curr. Biol. 28, 588–593. e4. (10.1016/j.cub.2018.01.012) 29429616

[67] Feord RC, Sumner ME, Pusdekar S, Kalra L, Gonzalez-Bellido PT, Wardill TJ 2020 Cuttlefish use stereopsis to strike at prey. Sci. Adv. 6, eaay6036 (10.1126/sciadv.aay6036) 31934631 PMC6949036

[68] Adams WJ, Graf EW, Anderson M 2019 Disruptive coloration and binocular disparity: breaking camouflage. Proc. R. Soc. B 286, 20182045 (10.1098/rspb.2018.2045) PMC6408597 30963917

[69] McKee SP, Watamaniuk SNJ, Harris JM, Smallman HS, Taylor DG 1997 Is stereopsis effective in breaking camouflage for moving targets? Vision Res. 37, 2047–2055. (10.1016/S0042-6989(96)00330-6) 9327053

[70] Nityananda V, O'Keeffe J, Umeton D, Simmons A, Read JCA 2019 Second-order cues to figure motion enable object detection during prey capture by praying mantises. Proc. Natl Acad. Sci. USA 116, 27 018–27 027. (10.1073/pnas.1912310116) PMC6936431 31818943

[71] Cronin TW, Bok MJ 2016 Photoreception and vision in the ultraviolet. J. Exp. Biol. 219, 2790–2801. (10.1242/jeb.128769) 27655820

[72] Church SC, Bennett ATD, Cuthill IC, Hunt S, Hart NS, Partridge JC 1998 Does Lepidopteran larval crypsis extend into the ultraviolet? Naturwissenschaften 85, 189–192. (10.1007/s001140050483)

[73] Majerus MEN, Brunton CFA, Stalker J 2000 A bird's eye view of the peppered moth. J. Evol. Biol. 13, 155–159. (10.1046/j.1420-9101.2000.00170.x)

[74] Church SC, Bennett ATD, Cuthill IC, Partridge JC 1998 Ultraviolet cues affect the foraging behaviour of blue tits. Proc. R. Soc. B 265, 1509–1514. (10.1098/rspb.1998.0465)

[75] Shashar N, Hagan R, Boal JG, Hanlon RT 2000 Cuttlefish use polarization sensitivity in predation on silvery fish. Vision Res. 40, 71–75. (10.1016/S0042-6989(99)00158-3) 10768043

[76] Johnsen S, Gagnon YL, Marshall NJ, Cronin TW, Gruev V, Powell S 2016 Polarization vision seldom increases the sighting distance of silvery fish. Curr. Biol. 26, R752–R754. (10.1016/j.cub.2016.07.030) 27554649

[77] Pignatelli V, Temple SE, Chiou T-H, Roberts NW, Collin SP, Marshall NJ 2011 Behavioural relevance of polarization sensitivity as a target detection mechanism in cephalopods and fishes. Phil. Trans. R. Soc. B 366, 734–741. (10.1098/rstb.2010.0204) 21282177 PMC3049012

[78] Hughes NK, Price CJ, Banks PB 2010 Predators are attracted to the olfactory signals of prey. PLoS ONE 5, e13114 (10.1371/journal.pone.0013114) 20927352 PMC2948037

[79] Geipel I, Steckel J, Tschapka M, Vanderelst D, Schnitzler H-U, Kalko EKV, Peremans H, Simon R 2019 Bats actively use leaves as specular reflectors to detect acoustically camouflaged prey. Curr. Biol. 29, 2731–2736.e3. (10.1016/j.cub.2019.06.076) 31378617

[80] Kalmijn AJ 1971 The electric sense of sharks and rays. J. Exp. Biol. 55, 371–383. 5114029 10.1242/jeb.55.2.371

[81] Ryer CH, Lemke JL, Boersma K, Levas S 2008 Adaptive coloration, behavior and predation vulnerability in three juvenile north Pacific flatfishes. J. Exp. Mar. Biol. Ecol. 359, 62–66. (10.1016/j.jembe.2008.02.017)

[82] Tinbergen L 1960 The natural control of insects in pinewoods. I. Arch. Néerl. Zool. 13, 265–336. (10.1163/036551660x00053)

[83] Bond AB, Kamil AC 2002 Visual predators select for crypticity and polymorphism in virtual prey. Nature 415, 609–613. (10.1038/415609a) 11832937

[84] Bond AB, Kamil AC 1999 Searching image in blue jays: facilitation and interference in sequential priming. Anim. Learn. Behav. 27, 461–471. (10.3758/BF03209981)

[85] Langley CM, Riley DA, Bond AB, Goel N 1996 Visual search for natural grains in pigeons (*Columba livia*): search images and selective attention. J. Exp. Psychol. Anim. Behav. Process. 22, 139–151. (10.1037/0097-7403.22.2.139) 8618099

[86] Stevens M, Marshall KLA, Troscianko J, Finlay S, Burnand D, Chadwick SL 2013 Revealed by conspicuousness: distractive markings reduce camouflage. Behav. Ecol. 24, 213–222. (10.1093/beheco/ars156)

[87] Troscianko J, Lown AE, Hughes AE, Stevens M 2013 Defeating crypsis: detection and learning of camouflage strategies. PLoS ONE 8, e73733 (10.1371/journal.pone.0073733) 24040046 PMC3769369

[88] Penacchio O, Harris JM, Lovell PG 2017 Establishing the behavioural limits for countershaded camouflage. Sci. Rep. 7, 1–11. (10.1038/s41598-017-13914-y) 29057907 PMC5651847

[89] Johnsen S 2014 Hide and seek in the open sea: pelagic camouflage and visual countermeasures. Annu. Rev. Mar. Sci. 6, 369–392. (10.1146/annurev-marine-010213-135018) 23987915

[90] Xiao F, Cuthill IC 2016 Background complexity and the detectability of camouflaged targets by birds and humans. Proc. R. Soc. B 283, 20161527 (10.1098/rspb.2016.1527) PMC5031667 27629039

[91] Dimitrova M, Merilaita S 2010 Prey concealment: visual background complexity and prey contrast distribution. Behav. Ecol. 21, 176–181. (10.1093/beheco/arp174)

[92] Hall JR, Cuthill IC, Baddeley R, Shohet AJ, Scott-Samuel NE 2013 Camouflage, detection and identification of moving targets. Proc. R. Soc. B 280, 20130064 (10.1098/rspb.2013.0064) PMC3619462 23486439

[93] Pan JS, Bingham N, Chen C, Bingham GP 2017 Breaking camouflage and detecting targets require optic flow and image structure information. Appl. Opt. 56, 6410–6418. (10.1364/AO.56.006410) 29047842

[94] Jabłoński PG 1999 A rare predator exploits prey escape behavior: the role of tail-fanning and plumage contrast in foraging of the painted redstart (*Myioborus pictus*). Behav. Ecol. 10, 7–14. (10.1093/beheco/10.1.7)

[95] Jabłoński PG, Lee SD, Jerzak L 2006 Innate plasticity of a predatory behavior: nonlearned context dependence of avian flush-displays. Behav. Ecol. 17, 925–932. (10.1093/beheco/arl039)

[96] Galatowitsch ML, Mumme RL 2004 Escape behavior of neotropical homopterans in response to a flush–pursuit predator. Biotropica 36, 586–595. (10.1646/1600)

[97] Shen Z, Neil TR, Robert D, Drinkwater BW, Holderied MW 2018 Biomechanics of a moth scale at ultrasonic frequencies. Proc. Natl Acad. Sci. USA 115, 12 200–12 205. (10.1073/pnas.1810025115) PMC6275474 30420499

[98] Dawkins R, Krebs JR 1979 Arms races between and within species. Proc. R. Soc. B 205, 489–511. (10.1098/rspb.1979.0081) 42057

[99] Kikuchi DW, Pfennig DW 2010 Predator cognition permits imperfect coral snake mimicry. Am. Nat. 176, 830–834. (10.1086/657041) 20950143

[100] Kikuchi DW, Mappes J, Sherratt TN, Valkonen JK 2016 Selection for multicomponent mimicry: equal feature salience and variation in preferred traits. Behav. Ecol. 27, 1515–1521. (10.1093/beheco/arw072)

